# Re: Concept and application of relaxing radial retinectomy for retinal detachment with advanced proliferative vitreoretinopathy

**DOI:** 10.1186/s40942-021-00303-x

**Published:** 2021-04-09

**Authors:** Anna Grabowska, James E. Neffendorf, Tom H. Williamson

**Affiliations:** 1 Department of Ophthalmology, King’s College Hospital NHS Foundation Trust, Denmark Hill, London, SE5 9RS UK; 2 Department of Ophthalmology, St Thomas’ Hospital, London, UK

## Abstract

The following is a response to the recent review article by Girsang and colleagues (Int J Retina Vitreous. 2020;6:46), who describe concept and application of relaxing radial retinectomy for retinal detachment with advanced proliferative vitreoretinopathy. We discuss the distribution of the retinal nerve fiber layer, an aspect not touched on by the authors, and the importance of its consideration in determining visual field outcomes when performing retinectomy. Moreover, we share our clinical experience with both radial and circumferential retinectomy and discuss scenarios where the combination of both is more effective.

Dear Editor,

We have read with interest the recent review article by Girsang et al. entitled “Concept and application of relaxing radial retinectomy for retinal detachment with advanced proliferative vitreoretinopathy” [1]. In this review, the authors describe the concept of radial relaxing retinectomy and propose radial relaxing retinectomies in 1–4 quadrants, considering the size and grade of proliferative vitreoretinopathy (PVR).

We would like to comment on some aspects of this review and take this opportunity to present our experience with circumferential and radial retinectomies.

Several studies have shown good reattachment rates using retinectomy in the treatment of rhegmatogenous retinal detachment complicated by advanced PVR [2, 3]. These studies describe the use of circumferential retinectomy. Furthermore, inferior PVR is a more common finding than superior PVR [4]. It has been noted that a circumferential retinectomy relieves anteroposterior retinal shortening, but is not effective in the management of circumferential shortening commonly found in more advanced stages of PVR [5].

The authors proposed radial retinectomies alone at 1, 5, 8, and 11 o’clock positions. They advocated that the cutting boundaries of the retinectomy are defined considering the topographic organization of the macula and should be located more than 6 mm away from the central fovea or outside the large vascular arcade surrounding the posterior pole. The authors do not mention the distribution of the retinal nerve fiber layer (RNFL), which is formed by the expansion of the fibers of the optic nerve (Fig. 1). This is an important aspect, as performing large retinectomies in the highest distribution of the RNFL may lead to significant visual field loss postoperatively.

**Fig. 1:**
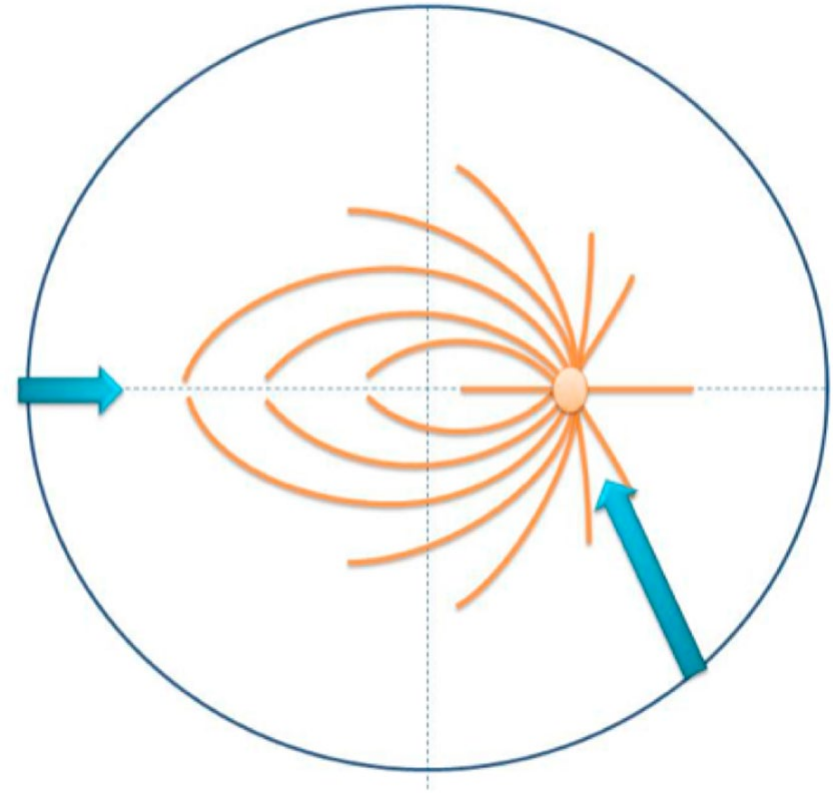
Nerve fiber layer distribution. Radial retinectomy should be performed in the direction of the arrows to minimize the number of the nerve fibers cut during the retinectomy thereby reducing visual field loss. Reprinted with permission from Williamson [6]

According to the authors, radial retinectomy should be performed in the most heavily wrinkled area of the retina or across the circumferential membrane formation. We believe that radial retinectomy alone may be ineffective in cases where peeling of the membrane proliferation at the vitreous base is not fully possible and the traction persists.

In our experience, the combination of both radial and circumferential retinectomy relieves circumferential shortening more effectively and prevents large visual field defects. In our clinical practice, we have noticed that there are two common scenarios in advanced PVR, which may require both radial and circumferential retinectomy [6].

The first common scenario is circumferential shortening of the retina, seen as folding of the retina inferior to the disc, often passing through the macula horizontally. In this case, radial retinectomy is performed between the inferotemporal and the inferonasal arcade, being cautious to avoid the major retinal blood vessels and cutting across too many retinal nerve fibers. Occasionally, a small retinectomy will be needed at the temporal upper end of the retinectomy (Fig. 2).

**Fig. 2:**
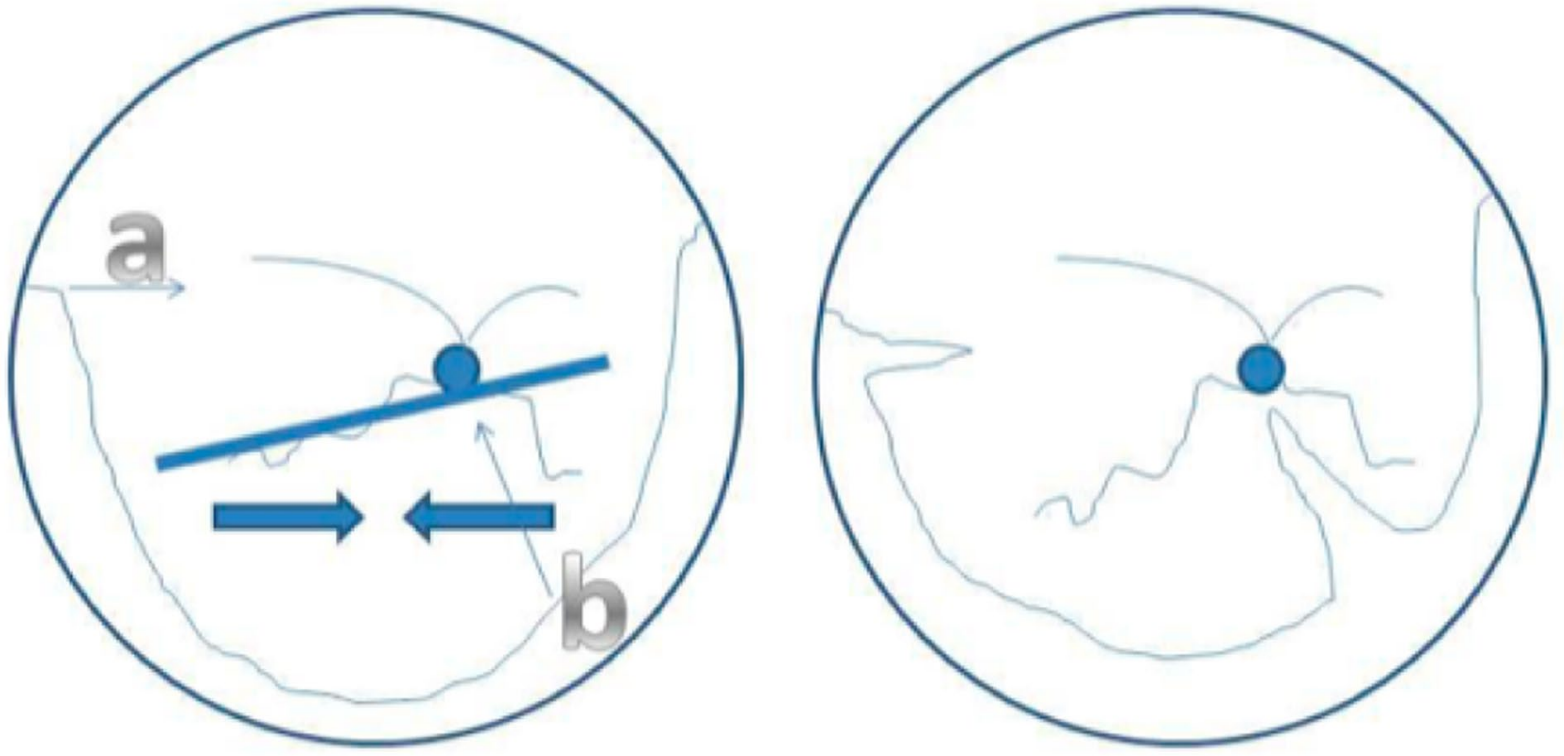
Location of radial retinectomies. After the circumferential retinectomy, a fold of the retina inferior to the optic disc indicates circumferential shortening (thick line and arrows). This can be relieved by performing a small radial retinectomy at (**a**) and a longer radial retinectomy at (**b**) to allow the retina to open like a flower petal. Reprinted with permission from Williamson [6]

The second most frequent situation is circumferential shortening at the edge of the previous retinectomy. In this case, a small circumferential retinectomy can be performed with a T-shaped radial cut in the middle of circumferential retinectomy (Fig. 3).

**Fig. 3:**
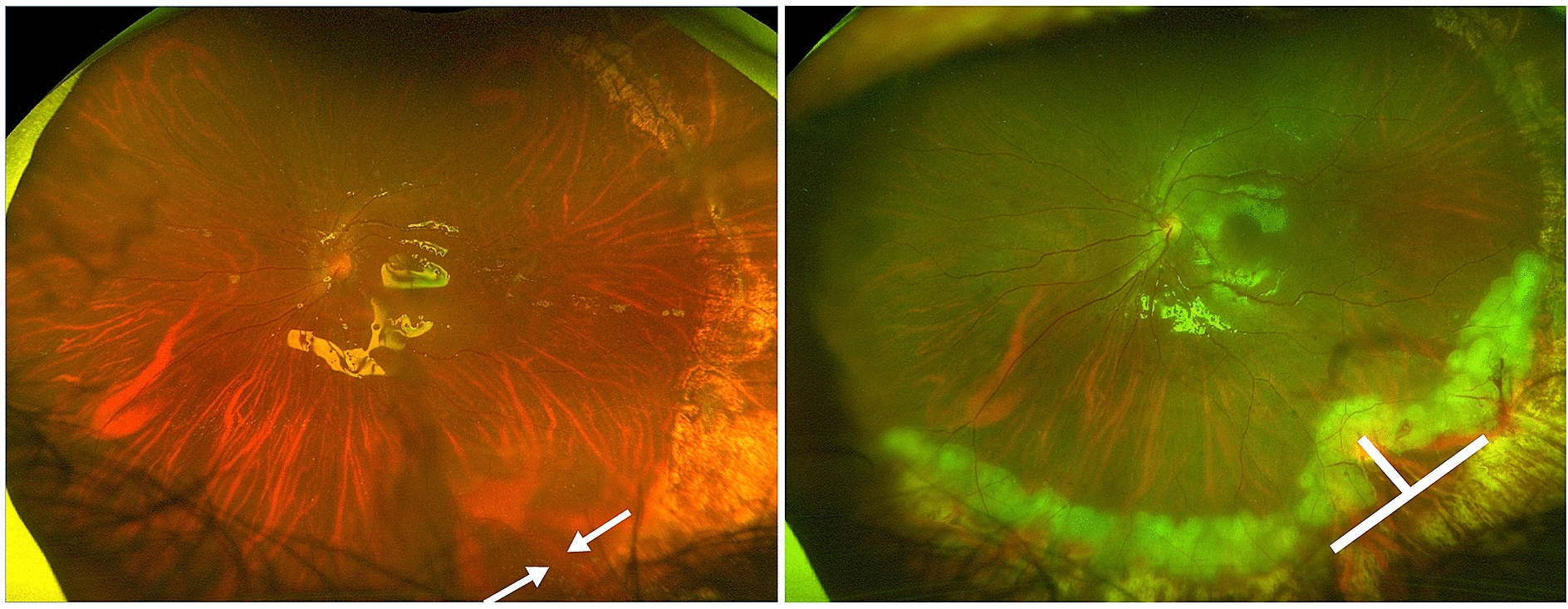
T-shape retinectomy. After giant retinal tear surgery, the area of subretinal fluid indicates circumferential shortening (left image; arrows). This is relieved by a T-shape circumferential and radial retinectomy (right image; thick lines)

In both these scenarios, care is taken to avoid cutting across retinal nerve fibers and large retinal vessels and to minimize the retinal excision. We believe that radial retinectomy is a good option alongside circumferential retinectomy when residual circumferential shortening cannot be fully eliminated. Further studies are needed to show if the combination of the two techniques is superior or inferior to radial or circumferential retinectomy alone.

Anna Grabowska

James E. Neffendorf

Tom H. Williamson

London, UK

## Data Availability

Not applicable.

## Abbreviations

PVR: Proliferative vitreoretinopathy
RNFL: Retinal nerve fiber layer
